# Epispadias in the Female

**Published:** 1887-03

**Authors:** 


Epispadias in the Female.—R. Dohon, in the *Zeitschrift f. Geb. and Gyn.*, xii., 1, reports this rare case: Patient æt 18 Has dribbling of urine Labia majora not prominent. Labia minora more prominent, and divided above, as also the clitoris This organ consisted of two symmetrical halves, and at the base of these halves, at the site of the normal junction above, was the urethra, wide enough to admit the little finger. The operation for relief consisted in dissecting off a three-cornered flap, the apex of which lay at the mons veneris, and the other two angles out beyond the two halves of the clitoris. The clitoris prepuce was freshened on each side, and sutured together. Good union followed, and the meatus lay in its normal position under the clitoris The girl improved slowly, and began to retain her urine, the tonicity of the vesical sphincter being stimulated by the use of electricity.

